# Diazotrophs and N_2_-Fixation Associated With Particles in Coastal Estuarine Waters

**DOI:** 10.3389/fmicb.2018.02759

**Published:** 2018-11-16

**Authors:** Jeppe N. Pedersen, Deniz Bombar, Ryan W. Paerl, Lasse Riemann

**Affiliations:** Marine Biological Section, Department of Biology, University of Copenhagen, Helsingør, Denmark

**Keywords:** diazotrophs, nitrogen fixation, marine particles, *nifH*, estuary

## Abstract

Putative heterotrophic bacteria carrying out N_2_-fixation, so-called non-cyanobacterial diazotrophs (NCDs), are widely distributed in marine waters, but details of how the O_2_-inhibited N_2_-fixation process is promoted in the oxic water column remains ambiguous. Here we carried out two experiments with water from a eutrophic temperate fjord to examine whether low-oxygen microenvironments within particulate organic matter could be loci suitable for N_2_-fixation. First, water enriched with natural particles or sediment showed higher N_2_-fixation rates than bulk water, and nitrogenase genes (*nifH*) revealed that specific diazotrophs were affiliated with the particulate matter. Second, pristine artificial surfaces were rapidly colonized by diverse bacteria, while putative diazotrophs emerged relatively late (after 80 h) during the colonization, and phylotypes related to *Pseudomonas* and to anaerobic bacteria became dominant with time. Our study pinpoints natural particles as sites of N_2_-fixation, and indicates that resuspension of sediment material can elevate pelagic N_2_-fixation. Moreover, we show that diverse natural diazotrophs can colonize artificial surfaces, but colonization by “pioneer” bacterioplankton that more rapidly associate with surfaces appears to be a prerequisite. Whereas our experimental study supports the idea of pelagic particles as sites of N_2_-fixation by heterotrophic bacteria, future *in situ* studies are needed in order to establish identity, activity and ecology of particle associated NCDs as a function of individual particle characteristics.

## Introduction

N_2_-fixation by bacterioplankton (diazotrophy) is an important external input of nitrogen to the ocean (Karl et al., 2002; Carpenter and Capone, 2008; Benavides and Voss, 2015). Besides cyanobacteria (Zehr, 2011), analyses of *nifH* genes show that non-cyanobacterial diazotrophs (NCDs) are almost ubiquitous and occasionally active in marine waters (Zehr et al., 1998; Riemann et al., 2010; Farnelid et al., 2011), but knowledge about their ecology and contribution to overall N_2_-fixation remains sparse (e.g., Bombar et al., 2016; Moisander et al., 2017).

One current enigma is how single-celled heterotrophic bacteria are able to fix N_2_ in the presence of dioxygen (O_2_), as it inhibits nitrogenase activity presumably due to the toxicity of O_2_ to the enzyme (Goldberg et al., 1987). While cyanobacteria avoid O_2_-inhibition by, e.g., heterocyst formation or temporal segregation of photosynthetic O_2_ production and N_2_-fixation (Reddy et al., 1993; Berman-Frank et al., 2007), little is known about the strategies employed by NCDs. Some non-marine NCDs protect nitrogenase by surrounding themselves by a layer of O_2_-impermeable extracellular polymers (Sabra et al., 2000) – and a somewhat similar strategy was recently observed in an oxygenated culture of a free-living marine *Pseudomonas* strain (Bentzon-Tilia et al., 2015a). Modeling, however, suggests that this strategy is energetically costly to the cell, even more than N_2_-fixation itself (Inomura et al., 2016), conceivably constraining the utility of this strategy among free-living bacterioplankton, especially in oligotrophic waters. Alternatively, heterotrophic bacteria may thrive and fix N_2_ in low-oxygen microenvironments associated with naturally occurring nutrient- and carbon rich particles (Ploug et al., 1997; Klawonn et al., 2015).

In the open ocean, most particles originate from the productive upper sunlit layer, while in coastal zones, resuspended sediment represents a greater share of all observed particles (Turner and Millward, 2002). Earlier research has shown that marine N_2_-fixation can be stimulated by natural and artificial particles (Guerinot and Colwell, 1985; Paerl and Carlton, 1988; Benavides et al., 2013; Rahav et al., 2013, 2016), that NCDs can colonize surfaces, e.g., copepod exoskeletons (Proctor, 1997; Braun et al., 1999; Scavotto et al., 2015), and that they are potentially symbionts of other plankton (Farnelid et al., 2010). Further, genomes and metagenomes of NCD’s frequently harbor genes encoding motility and chemotaxis (Delmont et al., 2018), enabling them to find and exploit particles, and most recently diverse NCDs were found on sinking marine particles in the North Pacific Subtropical Gyre (Farnelid et al., 2018). Thus, these studies collectively indicate that particles could be suitable loci for N_2_-fixation in marine waters.

In the present study, we carried out two experiments with water from the eutrophic Roskilde Fjord (RF, Denmark) to examine whether the presence of natural particles would stimulate N_2_-fixation by NCD’s, and whether NCD communities could efficiently colonize and proliferate on artificial surfaces. Our results overall support the hypothesis that particles can be important habitats for NCDs in marine pelagic waters.

## Materials and Methods

### Experimental Approach

In the first experiment, the “*particle enrichment experiment*,” we aimed to examine N_2_-fixation associated with natural particles (>100 μm diameter). N_2_-fixation rates, bacterial abundance, and diazotrophic community composition were compared among treatments with: (1) elevated particle concentration, (2) non-treated seawater (bulk), (3) filtrate (<100 μm), and (4) a suspension of top layer sediment in 100 μm filtered RF water. In the second experiment, the “*colonization experiment*,” artificial surfaces (see below) were incubated for 400 h in RF water. This experiment aimed to examine the colonization of newly formed particle surfaces by heterotrophic bacteria and diazotrophs under more controlled conditions.

### Water Sampling

Surface water (250 L) was collected on 11 April 2016 from “outer buoy” station (N 55° 55′ 26.05′′ E 12° 1′ 6.59′′) in RF using a rinsed bucket and acid-rinsed 50 L/25 L containers. At the time of sampling (10:00 UTC), the water temperature was 8.3°C, pH was 8.5, and salinity was 18.1 (multi-parameter Instrument Pro Plus, YSI). After the collection of seawater, samples from the sediment surface (ca. 50 cm^3^) were taken from ∼5 m depth using a mini van Veen grab sampler, and kept at ambient temperature until use.

### N_2_-Fixation Rate Measurements

**FIGURE 1:**
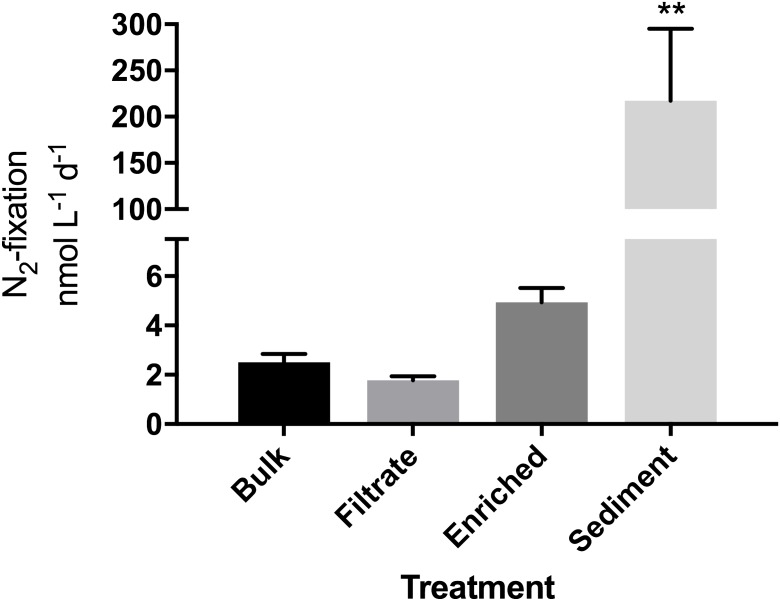
N_2_-fixation rates in the particle enrichment experiment measured in quadruplicate incubations. Due to a sample loss, an average of T0 values from the filtrate and enriched samples (*N* = 8) was used as the T0 value for the bulk sample. ^∗∗^Significantly different from all other (Tukey’s; *p* = 0.001).

N_2_-fixation was measured by ^15^N_2_-incorporation (Montoya et al., 1996). ^15^N_2_ tracer gas was predissolved in artificial seawater (salinity 17) as previously described (Mohr et al., 2010). First, 1.2 L polycarbonate incubation bottles were filled close to capacity and then aliquots of the tracered water were quickly added to incubations before immediately closing the bottles to obtain a theoretical initial ^15^N_2_ substrate label of ∼5 atom % ^15^N. The artificial seawater was prepared as previously described (Boström et al., 2007). Following 0.2 μm filtration, the artificial seawater was degassed for 1 h while applying heating (50°C), magnet stirring and vacuum, thereafter distributed into 50 mL borosilicate serum vials, which were immediately crimp sealed using butyl rubber stoppers. One milliliter of ^15^N_2_ tracer gas (Campro Scientific, Veenendaal, The Netherlands; 98% enrichment of ^15^N) was introduced into each vial using a gas-tight syringe, and the gas was dissolved at 5°C for at least 48 h. The batch of ^15^N_2_ gas from Cambridge Isotopes (lot # I-16727) was described to contain only minute levels of N contaminants, theoretically triggering maximal “false” rates of ca. 0.02 nmol N L^-1^ d^-1^ (Dabundo et al., 2014). Further, in order to avoid potential contamination with trace elements in this degassing protocol, all materials, bottles and tubings were acid-washed prior to use, and bottles were rinsed 3x with field water prior to filling. The minimum quantifiable rate (MQR) for each treatment was calculated using standard propagation of errors via the observed variability between replicate samples (Gradoville et al., 2017). The MQRs for treatments “bulk,” filtrate, particle enriched, and sediment resuspension were 1.1, 0.4, 0.8, and 86.9 nmoL N L^-1^ d^-1^, which are clearly lower than the rates observed in our experiment (see below and Figure 1).

### Particle Enrichment Experiment Methodology

For the “bulk” (untreated) treatment, water was filled into four 1.2 L polycarbonate bottles. To prepare for the remaining treatments, particles from 100 L of water were concentrated to 10 L using gentle reverse-filtration through a 100 μm pore size mesh within 1 h of sampling. The <100 μm water was used as the “filtrate” treatment, as well as for resuspending sediment (5 cm^3^ L^-1^) for the fourth treatment (sediment resuspension treatment to test for the role of sediment bacteria in pelagic N_2_-fixation). At T0, water from the four different treatments was transferred into quadruplicate 1.2 L polycarbonate bottles, and the bottles were topped off by adding one aliquot of 50 mL ^15^N_2_-enriched artificial seawater. For each of the four treatments, two additional replicate samples (no tracer added) were immediately filtered onto 25 mm pre-combusted (500°C, 12 h) glass fiber filters (Advantec, Toyo Roshi Kaisha, Japan, nominal pore size 0.3 μm) to obtain T0 natural abundance isotope measurements. All other (tracered) replicates were then incubated on a plankton wheel at ca. 2 rpm for 24 h at 8°C on a 14:10 h light: dark cycle (170 μmol photons m^-2^ s^-1^). Incubations were terminated by filtering 400–700 mL (40–60 mL for sediment incubation) of sample onto 25 mm pre-combusted (500°C, 12 h) glass fiber filters. All filters were kept at -80°C, followed by drying at 60°C for 20 h and pelletizing in tin cups (IVA, Meerbusch, Germany). Together with blank GF/F filters, all filters were analyzed by isotope ratio mass spectrometry for ^15^N and ^14^N at the Laboratory of Applied Physical Chemistry, Gent, Belgium.

### The Particle Enrichment Experiment: DNA Sampling

Three T0 and four T24 replicate water samples of 400–900 mL (100–160 mL for sediment treatment) were filtered onto 47 mm 0.2 μm Supor membrane filters (PALL Corporation, Port Washington, NY, United States) and stored at -80°C until nucleic acids extraction.

### Colonization Experiment Methodology

We hypothesized that after initial colonization and incipient remineralization on particles conditions will gradually become suitable for N_2_-fixation, and NCDs would proliferate. To test this hypothesis, we incubated “artificial particle surfaces” in RF water for a total of 400 h, with sampling points at 0, 15, 40, 80, 140, 210, 300, and 400 h. Particle surfaces were generated by dipping different filters (GF/F, polycarbonate and Supor^®^) into molten agarose (2%), forming a thin layer on the filter. The agar contained nutrients roughly at Redfield ratio with a starting organic carbon concentration of 2.5 mM (∼10× dissolved organic carbon *in situ*) (Redfield, 1934; Supplementary Table S1) to mimic fresh phytoplankton material. All filters were held in place between two plexiglass plates with a central circular outlet (66 × 66 × 5 mm; Supplementary Figure S1), placed randomly on strings, and incubated in a cylindrical tank containing 120 L of RF surface water at 8°C on a 14:10 h light:dark cycle (220 μmol photons m^-2^ s^-1^ surface white light). Water circulation was maintained using a hydropump with 1 L min^-1^ output. For each time point, triplicate 47 mm GF/F, polycarbonate (microscopy), and SUPOR filters (DNA) were sacrificed and analyzed. The GF/F filters served as samples for particulate organic carbon and nitrogen concentration measurements. GF/F filters were dried at 60°C for 20 h, pelleted in tin cups (IVA, Meerbusch, Germany), and measured on an isotope ratio mass spectrometer (PDZ Europa, Northwick, United Kingdom) at the Laboratory of Applied Physical Chemistry, Gent, Belgium together with ^15^N samples from the particle enrichment experiment. Bacteria were enumerated on black polycarbonate filters (Whatman, GE Healthcare, Little Chalfont, United Kingdom) using microscopy (see below). Abundances of free-living bacteria in water were determined at each time point by flow cytometry (see below). O_2_ profiles were measured on 3 random locations on GF/F filters down through the agar layer to the filter surface, using a FireStingO2, optical oxygen sensor (Pyroscience, Germany) mounted to a micromanipulator, and the lowest oxygen concentration at each position was noted. Oxygen concentration in the free water was also recorded using the optical sensor. Supor^®^ filters (0.22 μm pore size, Pall Corporation, Ann Arbor, Michigan, United States) served as samples for DNA based analysis of bacterial (16S rRNA genes) and diazotrophic (*nifH* genes) community composition. At each sampling point, these filters were removed from the filter holders and stored in 2 mL Eppendorf tubes at -80°C until extraction. In order to determine bacterial community composition in the surrounding water, triplicate 500 mL aliquots of water were at each time point filtered onto Supor^®^ filters and stored at -80°C.

### Bacterial Abundance Measurements

In the particle enrichment experiment, bacterial abundances were determined by microscopy. For each sample, 15 mL water were fixed (1% glutaraldehyde, final concentration) for 15 min. Bacteria were detached from particles by adding 10 μL of TWEEN80 (Riemann et al., 2000) followed by sonication for 30 s at 50 W (Vibracell VC50T). Cells were stained using SYBR green I (1% final concentration; Noble and Fuhrman, 1997) and 1–5 mL were filtered onto 0.22 μm black polycarbonate filters (Whatman, GE Healthcare, Little Chalfont, United Kingdom). Examination of successful bacterial detachment from particles and counting was done at 1,000× magnification on an Olympus BX61 microscope (DP70 camera), using a phenylenediamine mounting solution (Noble and Fuhrman, 1997).

For the colonization experiment, bacteria growing on the agar coated filters were fixed by covering the filter with 800 μL of glutaraldehyde (1% final) for 15 min. The fixative was then carefully removed with a pipette, and filters were air-dried for 15 min. Cells were stained with SYBR green I and >200 bacteria filter^-1^ or >15 fields filter^-1^ were counted at 1,000× magnification. To determine bacterial abundance in water, triplicate 2 mL aliquots were fixed with glutaraldehyde (1% final) and stored at -80°C. After defrosting, samples were stained with SYBR green I and analyzed on a FACSCanto II flow cytometer (BD Biosciences, Franklin Lakes, NJ, United States) according to (Gasol and del Giorgio, 2000).

### DNA Extractions

All samples were extracted using a phenol/chloroform protocol (Boström et al., 2004), quantified (PicoGreen, Molecular Probes, Invitrogen, Eugene, OR, United States), and kept at -80°C until PCR amplification.

DNA extractions were performed on triplicate T0 samples and quadruplicate T24 samples from all four treatments, yielding a total of 28 samples from the particle enrichment experiment. For the colonization experiment, DNA was extracted from triplicate T0 filters covered with Agarose (T0 water samples were the same as the T0 bulk samples from the particle enrichment experiment) and triplicate filter and water samples from another five chosen time points (T40, 80, 140, 300, 400), yielding another 33 samples in total. Extracts from the two T0 Agarose filters as well as further two blank filters were included in PCRs and Illumina sequencing, but did not produce visible bands in gel electrophoresis, and recovered sequences were of wrong size and only matched various non-target sequences in BLAST runs.

### PCR Amplification of *nifH* and 16S rRNA Genes

Amplification of *nifH* genes was attempted for all 28 samples from the particle enrichment experiment. For the colonization experiment, filters and water DNA samples from six time-points were analyzed for both 16S rRNA and *nifH* gene composition. PCR reactions were prepared in a UV workstation, and DNA templates were added in a separate UV workstation. The *nifH* genes were amplified using a nested PCR approach (Zani et al., 2000; Zehr and Turner, 2001) and primers with sample specific barcodes. The 16S rRNA genes were amplified using barcoded primers 515f and 806r (Caporaso et al., 2012) and MyTaq polymerase (Bioline, London, United Kingdom). For primers and barcodes, see Supplementary Table S2. For each gene, pooled triplicate PCR reactions for each sample were purified (Agencourt AMPure XP kit, Beckman Coulter, Indianapolis, United States) and quantified (PicoGreen). All samples were pooled in equimolar amounts (∼10 ng DNA per sample) and sequenced using Illumina technology (MiSeq v2 2 × 250 bp; NGI Sweden). Sequences were uploaded to the Sequencing Read Archive (SRA) database on NCBI (Accession number SRP158049).

### Bioinformatics

Using Qiime (Caporaso et al., 2010), raw assembled sequences were trimmed of overhangs and primers, and thereafter demultiplexed using sample specific barcodes. For the colonization experiment, *nifH* and 16S rRNA gene sequences were first separated using an amplicon size cutoff of 290 bp (filter_fasta script; Afgan et al., 2016), and potential misplacement of 16S rRNA or *nifH* genes was later excluded by BLASTing all major operational taxonomic units (OTUs) against the nr database in NCBI using Geneious v7.05. OTUs were clustered at 97% similarity using USEARCH (Edgar, 2010). For 16S rRNA gene sequences, taxonomy was assigned via the Silva database (Quast et al., 2013). Translated n*ifH* sequences were assigned to canonical clusters I-IV and subclusters (Zehr et al., 2003) using a classification and regression tree script (Frank et al., 2016). OTUs with <20 reads across all samples, or OTUs occurring in only one sample with <100 reads were removed. One T0 “bulk” sample from the particle enrichment experiment was excluded from further analysis because 92% of all reads clustered into only one OTU, likely pointing to PCR bias (Polz and Cavanaugh, 1998). Unfortunately, some samples were not PCR amplifiable and a variable number of replicates are therefore reported.

### Statistical Analysis

For non-metric multidimensional scaling (NMDS) plots, heatmaps, and statistical analyses, rarefied OTU tables were generated by subsampling all samples to the lowest read number in a single sample (5,311 reads, single_rarefaction.py in Qiime). For NMDS plots, Bray Curtis dissimilarity matrixes were calculated using PRIMER 6. Heatmap plots were constructed using ggplot2 in R (R Core Team, 2014). Further analyses were done in GraphPad Prism v. 6 (Prism; La Jolla, CA, United States) after testing data for requirements in R (Shapiro-Wilk normality test; R Core Team, 2014). In the particle enrichment experiment, for bacterial absolute abundance, a two-way ANOVA with Šidák’s correction was done while one-way ANOVA with Turkey’s multiple comparisons test was used to compare N_2_-fixation rates. Significance between treatments and controls was analyzed using *t*-tests as were linear and non-linear fits of data. *P*-values < 0.05 were considered significant.

## Results

### Particle Enrichment Experiment

#### Bacterial Abundances and Rates of N_2_-Fixation

Bacterial abundance did not differ between bulk water, particle-enriched, and filtrate treatments despite the 10-fold enrichment with particles (*p* = 0.1869), but was overall slightly lower after the 24 h incubation (*p* = 0.0196, Supplementary Figure S2). N_2_-fixation was measurable in all four treatments. Rates appeared higher in the enriched treatment compared to the filtrate (4.9 vs. 1.7 nmol N L^-1^ d^-1^; Figure 1), but this was not significant. While bacterial abundances were not significantly elevated in the sediment treatment (Supplementary Figure S2), the addition of sediment enhanced N_2_-fixation significantly (*p* = 0.001) to 217 nmol N L^-1^ d^-1^.

#### Diazotroph Community Composition

**FIGURE 2:**
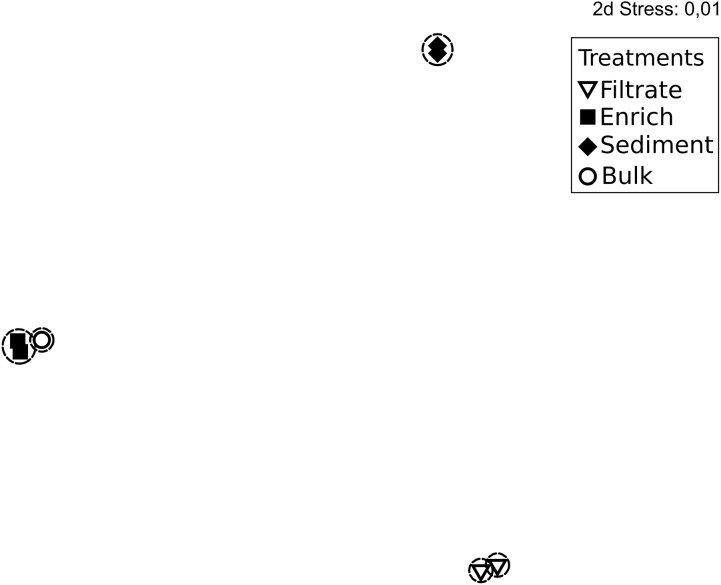
Non-metric multidimensional scaling plot based on rarefied (*n* = 1,680) *nifH* genes and Bray Curtis similarity of *nifH* gene composition in the particle experiment at T0. Encircled treatments have similarities >35%. Stress = 0.01. In some cases, only two replicates per sample are shown since amplification was unsuccessful for single replicates.

The 287,000 *nifH* sequences remaining after quality screening clustered into 476 OTUs. During incubation the communities clearly changed (Supplementary Figure S3). At T0, the *nifH* gene composition differed between treatments, with the bulk community falling between enriched, sediment and filtered samples (Figure 2). This pattern was supported by Morisita Horn (MH) indices (0 more similar – 1.0 not at all similar; Magurran, 2005). These are not affected by sample size (O’Keeffe, 2004), and therefore based on raw relative OTU abundances (Table 1). There was higher similarity between replicates in the enriched (MH 0.26) and sediment treatments (MH 0.66) compared to the filtrate (MH 0.93). Between treatments, the bulk was more similar to enriched (MH 0.75), while there was a low similarity between all other treatments (MH > 0.95).

**Table 1:** Morisita-Horn dissimilarity indices for *nifH* gene composition in treatments of the particle experiment; from 0 (identical) to 1 (completely different).

	Between replicates	Bulk	Filtrate	Enriched
Bulk		0		
Filtrate	0.930	0.985	0	
Enriched	0.258	0.752	0.992	0
Sediment	0.665	0.950	0.993	0.989

*The second column shows the difference between replicates; no value is noted for only one successfully amplified bulk replicate. The values given in columns 3–5 are based on comparing averages calculated from all available replicates within single treatments.*

**FIGURE 3:**
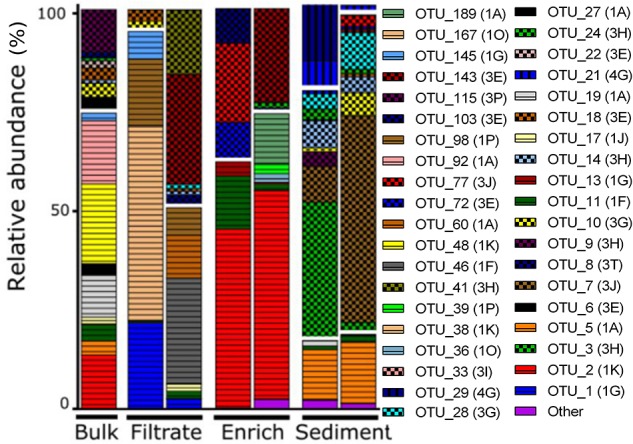
*NifH* gene composition in the particle experiment at T0. Operational taxonomic units (OTUs) shown each account for >2% of total read abundances in any sample. *NifH* gene clusters are split by horizontal white lines and marked by horizontal lines (Cluster I), checkered pattern (Cluster III), or vertical lines (Cluster IV). *NifH* subclusters according to Zehr et al. (2003) are indicated and the phylogenetic assignments are: 1A (Alpha-Proteobacteria); 1F (Epsilon-Proteobacteria); 1G and 1O (unassigned); 1J, 1K, and 1P (Beta-Proteobacteria); Subclusters of *nifH* Cluster III are putative anaerobic bacteria; Subclusters of *nifH* gene Cluster IV are from Archaea and distantly related chlorophyllide reductase genes. BLAST hits for predominant OTUs are shown in Supplementary Table S3. Only two replicates per sample are shown since amplification was unsuccessful for single replicates.

OTUs belonged mainly to *nifH* Clusters I (53% of all) and III (44% of all), while Clusters II and IV accounted for only 0.01 and 2% of all sequences, respectively (Figure 3). Sediment samples were dominated by *nifH* Cluster III, while the other treatments were dominated by Cluster I type sequences. Cluster IV sequences were exclusively found in sediment. The most dominant OTU 2 (accounting for 12% of all *nifH* reads) was only found in bulk and enriched treatments and was closely related to a strictly anaerobic *Alcaligenaceae* bacterium (99.1% nucleotide similarity; Supplementary Table S3). OTU 1 was mainly present in the filtrate (only 0.01–0.12% read abundance in bulk and enriched treatments, respectively) and is identical to BAL376, which is a *Pseudomonas*-like diazotroph isolated from the Baltic Sea (Supplementary Table S3; Farnelid et al., 2014). Sediment samples were dominated by Cluster III sequences including OTU 7 (99.8% nucleotide similarity to microbial mat sequence GU193899; Supplementary Table S3) and OTU 3, which had only 84% nucleotide similarity to sequence AF216899, recovered from cordgrass rhizosphere (Lovell et al., 2000).

### Colonization Experiment

#### Bacterial Abundance and Colonization

**FIGURE 4:**
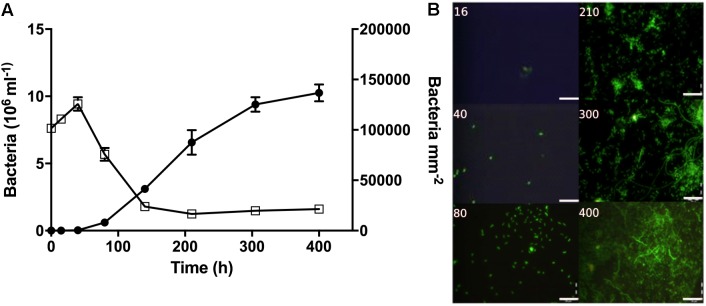
Bacterial abundance and morphology over time during the colonization experiment. **(A)** Bacterial abundance on filter surfaces (circles; cells mm^-2^) and in the surrounding water (open squares; cells × 10^6^ mL^-1^). **(B)** Representative pictures showing bacterial growth on the filter surfaces. Time (h) is noted on each picture. Scale bars = 20 μm.

During early colonization, a few bacteria were observed on the filter surfaces (Figure 4). After 80 h, bacterial abundances increased exponentially (Supplementary Table S4) and micro-colonies became visible. In the surrounding water, bacterial abundance peaked after 40 h at nearly 10 × 10^6^ cells mL^-1^, and then decreased to around 2 × 10^6^ cells mL^-1^ (Figure 4).

#### Succession in Community Composition on Filters

**FIGURE 5:**
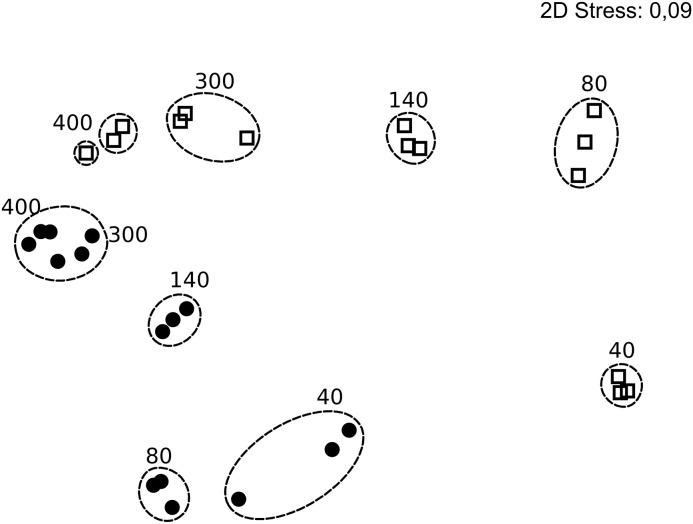
Non-metric multidimensional scaling plot of bacterial community composition in the colonization experiment over time, based on rarefied (*n* = 5,311) 16S rRNA genes and Bray Curtis similarity. Circles and squares represent filter and water samples, respectively, with time points (h) above the symbols. Dotted rings mark similarities >80% and include replicates. Stress 0.09. In some cases, less than the maximum number of replicates per sample is shown since amplification was unsuccessful for single replicates.

A total of 816,000 quality-screened 16S rRNA gene reads were obtained from triplicate water and filter samples at 5 time points in the colonization experiment, constituting 471 OTUs. Extensive community succession was observed over time, both on filters and in the water (Figure 5). Filter- and free-living communities differed, but this difference decreased over time (MH dissimilarity 0.65 at 40 h and MH 0.32 at 400 h; Supplementary Table S5), and there was only minor succession in both phases between 300 and 400 h (Figure 5).

**FIGURE 6:**
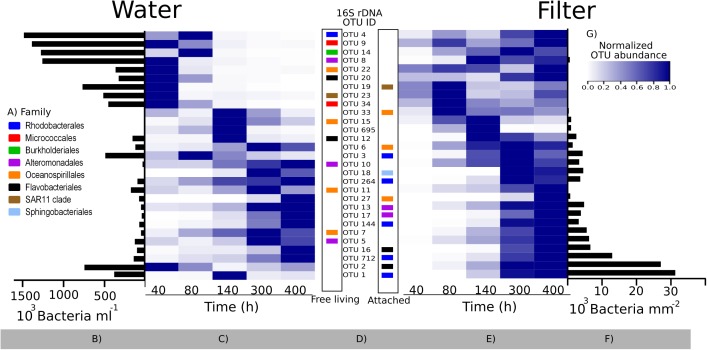
Bacterial community succession over time in the colonization experiment based on 16S rRNA gene sequencing. Relative abundances of the 29 most predominant operational taxonomic units (OTUs; representing >80% of reads in one or more samples) were normalized by multiplying with total bacterial abundance in each sample (see text). **(A)** Family taxonomy key; **(B,F)** maximum abundances of each OTU during the experiment in the surrounding water and on filters, respectively; **(C,E)** heatmaps showing each OTU normalized to its maximum abundance **(B,F)**; **(D)** OTU IDs corresponding to the heat map lines, with the color code showing their taxonomy. The placement of the colored squares indicates the isolation source of its closest marine BLAST hit (left = free living; right = attached to surfaces; see also Supplementary Table S5). **(G)** Color key for heatmaps. Note the difference in scale and unit for **(B,F)**.

An appropriate interpretation of bacterial community succession requires normalization of relative OTU abundances, in order to account for the considerable temporal changes in bacterial abundances over time (Figure 4A). This may be done by multiplying relative OTU abundances with total bacterial abundances in a given sample (Andersson et al., 2010). To visualize our data, we first depicted the 29 most predominant OTUs (representing >80% of reads in one or more samples). These OTUs belonged to eight different families (Figure 6A), with closely related sequences reported previously as either free-living or particle-attached (Figure 6D). Next, the maximum abundance over the entire course of the experiment for each OTU was determined (Figures 6B,F). Changes in abundance over time for each OTU were then plotted in heat maps as relative to its maximal abundance (Figures 6C,E).

Bacterial communities in the water were initially comprised of a few dominant OTUs, while after 80 h, a total of 20 lower-abundant OTUs were present (Figures 6B,C). After 40 h, only a few OTUs colonizing filters were detectable, including OTU 33, sharing an identical 16S rRNA sequence with a surface colonizing *Oceanospirillales* sp. from temperate coastal marine waters (Dang et al., 2008; Supplementary Table S6). However, after peaking in abundance at 80 h, relative abundances of OTU 33 declined. Major OTUs after 80 h included OTU 2, peaking at 300 h, and OTU 1, which succeeded after 300 h. Closest relatives of both OTUs were surface associated strains. OTU 2 belongs to the *Rhodobacteraceae* family, and was identical to a biofilm forming *Rhodobacterales* from an estuarine colonization experiment (LT549343, Supplementary Table S6). OTU 1 belongs to the *Flavobacteriaceae* family and is closely related to uncultured *Flavobacteriaceae* known to colonize macroalgae (99%, HM437512). Overall, there was a tendency for OTUs with an attached lifestyle (e.g., OTUs 1, 2, 16, and 712) to dominate the filters as bacterial abundance on filters increased over time (Figure 6E). Notably though, some OTUs (e.g., OTU 695) had only a transient appearance on filters.

**FIGURE 7:**
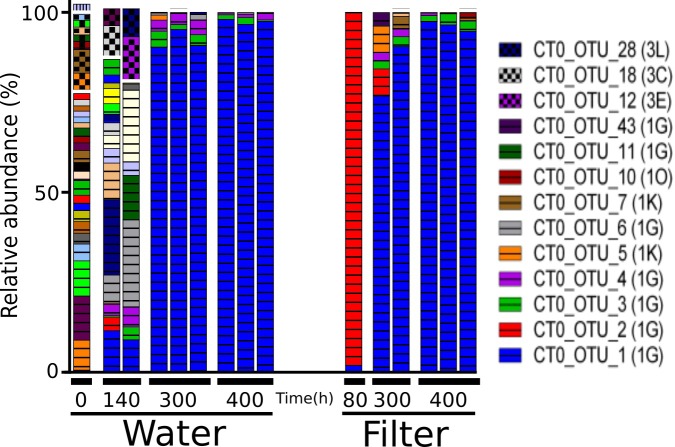
Relative abundance of the main *nifH* gene based operational taxonomic units (OTUs) each accounting for > 1% of reads in any sample in the colonization experiment. Only selected OTU labels are shown. *NifH* gene clusters are marked by horizontal lines (Cluster I) and checkered pattern (Cluster III), and vertical lines (Cluster IV). *NifH* subclusters according to Zehr et al. (2003) are indicated and the phylogenetic assignments are: 1G and 1O (unassigned); 1K (Beta-Proteobacteria); Subclusters of *nifH* Cluster III are putative anaerobic bacteria. BLAST hits for predominant OTUs are shown in Supplementary Table S3. In some cases, less than the maximum number of replicates per sample is shown since amplification was unsuccessful for single replicates.

*NifH* genes were successfully sequenced from 6 colonized filters and 9 samples of the surrounding water. The 651,000 sequences clustered into 98 OTUs. Forty OTUs accounting for >1% in any sample, collectively representing 93–99% of all reads per sample, were selected for further analysis (Figure 7). Initially, water samples harbored a diverse assemblage of *nifH* OTUs, but after 140 and 300 h only few OTUs were discernible, and thereafter *nifH* composition remained relatively stable (MH 0.003; Supplementary Table S7). At 80 h, one filter was colonized by CT0_OTU 2, which is identical to the OTU 1 found in filtrate samples in the particle enrichment experiment (Figure 3 and Supplementary Table S4). At 300 h, CT0_OTU 5 occurred, which has 99.1% nucleotide similarity to an anaerobe phylotype belonging to *Alcaligenaceae* (Supplementary Table S4). CT0_OTU 1 became dominant both in water and on filters after 300 h. A closely related OTU has previously been found in the ETSP (99% nucleotide similarity, HM801708; Fernandez et al., 2011), but these environmental sequences lack closely related cultivated representatives.

## Discussion

### Role of Natural Particles in Pelagic N_2_-Fixation

N_2_-fixation is at times stimulated by particle addition and N_2_-fixation rates can be positively correlated with the sizes and concentrations of particles (Rahav et al., 2013; Benavides et al., 2015). Natural particles, e.g., originating from atmospheric dust, are also thought to enhance N_2_-fixation rates (Foster et al., 2009; Benavides et al., 2013). Thus, the original idea of particles as potential hotspots for N_2_-fixation (cf. Paerl and Carlton, 1988) has been persistent and was reiterated more recently (Riemann et al., 2010; Bombar et al., 2016). However, progress has been limited regarding the clear identification of diazotrophs associated with particles, and the precise abiotic conditions that trigger these NCDs to seek and exploit the particle microenvironment.

Our study shows that a 10-fold enrichment with natural particles (>100 μm) caused elevated N_2_-fixation compared to untreated or filtered seawater in the RF. The measured rates fall well within the range measured throughout an annual cycle in RF (0–45 nmol N L^-1^ d^-1^), with the highest rates occurring in April 2012 after a spring phytoplankton bloom (Bentzon-Tilia et al., 2015b). Our results, and the fact that half of the N_2_-fixation measured in spring 2012 was in the >10 μm size fraction (Bentzon-Tilia et al., 2015b), indicate that particle-associated activity can be responsible for a significant share of total N_2_-fixation in the eutrophic and shallow RF.

The low similarity between filtrate and enriched treatments (MH: 0.99; Table 1) suggests that certain heterotrophic diazotrophs are selectively thriving members of the particle community. Comparable data are scarce, but consistent with our findings, some diazotrophs (mainly γ-Proteobacteria such as γ-24774A11) appear almost exclusively in size fractions >3 μm (Benavides et al., 2016). Future molecular analyses of individually picked particles, instead of size-fractionated samples, could easily test the hypothesis that select NCD’s optimally grow and/or fix N_2_ on particles. See also the recent publication by Farnelid et al. (2018).

We did not examine the size spectrum of particles >100 μm in our experiment. Assuming that diazotrophs require suboxic to anoxic conditions in order to fix N_2_, particles would need to be at least around 1 mm in size for sub/anoxic centers to occur if the surrounding water is fully oxygenated (Ploug et al., 1997; Klawonn et al., 2015). However, the presence of certain bacteria can indicate whether suboxic or anoxic conditions occur (Simon et al., 2014; Stief et al., 2016). It is therefore noteworthy that the filtrate and bulk treatments harbored lower relative abundances of Cluster III *nifH* sequences compared to the particle-enriched, and especially, the sediment treatment. Most of the Cluster III *nifH* genes were related to phylotypes reported earlier for sediments or mudflats, for example OTU 24, a phylotype also found in a temperate New England estuary (accession number KF662300; Newell et al., 2016). Since Cluster III mainly contains anaerobic bacteria (Raymond et al., 2004), our data collectively indicate that suboxic to anoxic zones were associated with natural particles >100 μm, and that resuspension of sediment material to some extent contributes to pelagic particles in the RF. Interestingly, the diazotroph communities in the enriched and the sediment treatments had limited overlap (MH 0.99; Table 1), suggesting that resuspended sediment was not a main source of pelagic particles at the time of sampling. However, in a previous RF study (Bentzon-Tilia et al., 2015b), diazotrophs affiliated with Cluster III occasionally reached high relative abundances in pelagic samples, suggesting occasionally extensive sediment resuspension probably driven by wind-induced mixing. Additionally, since these Cluster III NCD’s remained post-particle enrichment (>100 μm or sediment) it seems their particle associations are more robust than fragile.

N_2_-fixation rates as well as specific uptake of N (*V*) (Montoya et al., 1996) in the resuspended sediment treatment were clearly higher than in the other treatments reaching up to 217 nmol N L^-1^ d^-1^. Such rates are also higher than those previously reported for RF (up to 47 nmol N L^-1^ d^-1^; Bentzon-Tilia et al., 2015b). While our experimental approach may be extreme, and the resulting N_2_-fixation too high relative to natural conditions, it does indicate that resuspension of sediment material may increase pelagic N_2_-fixation. Estuarine sediments are often considered net nitrogen sinks due to denitrifying bacteria thriving in the anoxic layers (Fulweiler et al., 2007), however, together with other recent studies (Fulweiler et al., 2013; Fan et al., 2015), our data suggest that significant N_2_-fixation can take place in the sediment as well as occasionally when sediment material is resuspended in the water column. Indeed, sediment resuspension may decrease the particulate N:P ratio in the water column (Holmroos et al., 2012) and this may stimulate N_2_ fixation activity (Knapp, 2012).

### Colonization of Artificial Particle Surfaces

If marine particles are important sites for N_2_-fixation by NCDs, then these bacteria are likely adapted for colonizing surfaces. The colonization experiment aimed at testing this by monitoring diazotroph community succession and oxygen concentrations within artificial particle surfaces. The colonization experiment, based on incubation of a confined water mass over time, carried the inherent risk of a “bottle effect” where confinement affects bacterial community composition via opportunistic growth by some taxa and disappearance of others (e.g., Eilers et al., 2000). Indeed, the observed decrease in bacterial abundance in the water phase as well as the pronounced bacterial community succession is likely evidence of a bottle effect. Moreover, we applied model surfaces (GF/F, polycarbonate and Supor filters) that represent hydrophobic and hydrophilic materials expected in nature, however, they are likely different from natural particle surfaces (in heterogeneity, surface charge, etc.), and even differ from each other due to methodological necessity. Taken together such potential biases prevent direct extrapolation of our findings to natural environments. Nevertheless, the experiment illustrates a succession of bacterial phylotypes on the artificial surfaces from being mainly known free-living taxa to becoming dominated by known surface associated taxa. Further, that diazotrophs appeared as secondary colonizers of these surfaces. While these dynamics may have been affected by the confinement effect inherent with any batch experiment, they are consistent with the fact that natural marine particles are enriched with taxa known to be found in association with particles (Farnelid et al., 2018) and the appearance of secondary consumers at later stages of surface colonization (Datta et al., 2016). Hence, we speculate that NCD’s in nature are secondary colonizers of newly formed particles – likely due to their dependence on conditions established by pioneering populations and/or initial antagonisms within the particle microenvironment. Future experiments performed *in situ* could test the validity of these ideas and yield insight into the identities of key diazotrophic taxa colonizing and proliferating on marine particles in the wild.

Surprisingly, we did not detect low-oxygen zones on the filters using microelectrode measurements. This was likely explained by the patchy colony formation on the filters (Figure 4B), and limited capacity to carry out multiple point measurements across single filters using the optical sensor. Bacteria were observed on filters after 16 h, and 16S rRNA gene sequencing showed the presence of OTUs associated with non-diazotrophic associated taxa *Rhodobacterales*, *Alteromonadales*, and *Oceanospirillales* after 40 h. OTU 33 was among the first colonizers, and its relative abundance increased until 80 h. Interestingly, OTU 33 is identical to uncultured bacteria (EF215802) found among the first colonizers of artificial surfaces in coastal waters of the West Pacific Ocean (Dang et al., 2008). OTU 2 (*Flavobacteriales*) sequentially succeeded as a dominant bacterium, and ultimately OTU 1 (*Rhodobacterales*). *Flavobacteriaceae* and *Rhodobacteraceae* have also been found to colonize artificial chitin particles (Datta et al., 2016) and were dominant on particles in coastal ecosystems (Simon et al., 2014; Mestre et al., 2017). Hence, the abundant 16S rRNA OTUs in this experiment belong to bacterial groups specialized in colonizing marine particles.

Successful amplification of *nifH* genes from filters was achieved only after 80 h of incubation, suggesting NCD’s as “secondary” or “tertiary” colonizers of newly available marine particles – following pioneering taxa like OTUs 33 and 19 (Figure 6 and Supplementary Table S6). Presumably conditions created by pioneering taxa facilitated the recruitment of select NCD’s in our experiment; although speculative, this may include chemical modifications within the particle microenvironment, e.g., polysaccharide generation, infochemicals, or hydrolysis products. In addition, pioneering species likely facilitate conditions suitable for N_2_ fixation, including suboxia or anoxia. Indeed, recent work indicated that aggregation of cells or an actively respiring biofilm were conditional for particle colonization and N_2_-fixation by a cultivated heterotrophic bacterium (Paerl et al., 2018). Initially, *nifH* was amplifiable from only one replicate, and retrieved sequences clustered almost exclusively with CT0_OTU 2. This OTU was 99% identical to OTU 1 from the particle enrichment experiment (*Pseudomonas*-like Gammaproteobacterium BAL376; Farnelid et al., 2014), where it, however, mainly occurred in the filtrate treatment. This organism was previously found to be a predominant pelagic diazotroph in the RF after the spring bloom (10^4^ transcripts L-1; Bentzon-Tilia et al., 2015b) and it has previously been detected in the filtrate of 0.7 μm pore size filters (Bombar et al., 2018). Hence, it seems that BAL376 can alter between free-living and particle–associated lifestyles. In concert with previous work, this study pinpoints *Pseudomonas* as a key taxon to heterotrophic N_2_-fixation in the Baltic region – possibly with even wider importance as it has been found in marine surface waters around the world (Church et al., 2005; Foster et al., 2009; Farnelid et al., 2011; Fernandez et al., 2011; Bentzon-Tilia et al., 2015b). Toward the end of the incubation, CT0_OTU 1 dominated both in water and on filters. This *nifH* OTU was 99% identical to diazotrophs previously recovered from ocean water, including anoxic habitats (Fernandez et al., 2011), and it was also found in the enriched and bulk treatments in the particle enrichment experiment (Figure 3).

## Conclusion

In conclusion, the present study supports that significant coastal water column N_2_-fixation can be associated with increases in “autochthonous” and “allochthonous” (sediment) water-column particles. In particular, our findings suggest a potentially important role for sediment resuspension in influencing water-column N-fixing rates in coastal systems. We also document that diverse NCDs have the ability to colonize artificial surfaces, and that extensive succession and proliferation of specific phylotypes occurs over time. Details of these interactions require further investigation based on *in situ* data, but inherently impact the potential for particle-localized N_2_-fixation.

## Author Contributions

JP, DB, and LR designed the study, and JP and DB carried out sampling and experiments. RP helped out with experimental work. JP wrote the first manuscript draft, which was then revised by all authors.

## Conflict of Interest Statement

The authors declare that the research was conducted in the absence of any commercial or financial relationships that could be construed as a potential conflict of interest.
