# Supplementary material for: Diazotrophs and N2-Fixation Associated With Particles in Coastal Estuarine Waters
Source: Front Microbiol. 2018 Nov 16;9:2759. doi: 10.3389/fmicb.2018.02759 (PMC6250843; doi:10.3389/fmicb.2018.02759)
Supplement: Supplementary file 1 [file Table_1.pdf]

Supplementary Material

Diazotrophs and N_2-fixation associated with particles in coastal estuarine waters

Jeppe N. Pedersen, Deniz Bombar*, Ryan W. Paerl[1] and Lasse Riemann*

Marine Biological Section, Department of Biology, University of Copenhagen,

Helsingør, Denmark

*** Correspondence:**
Deniz Bombar, Lasse Riemann
Email: dbombar@sund.ku.dk; lriemann@bio.ku.dk

Supplementary Figures

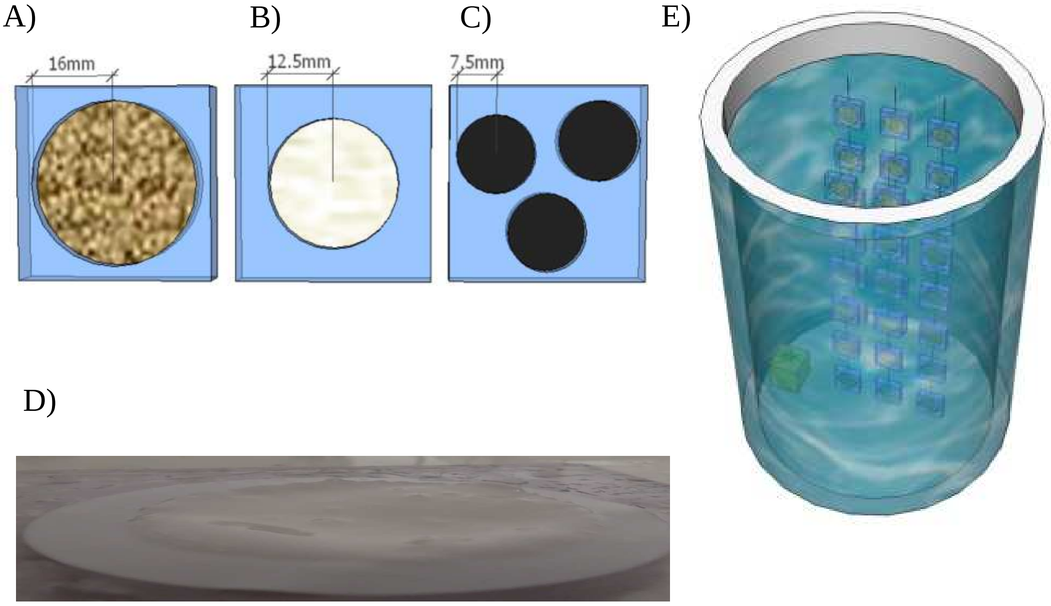

Fig. S1: Colonization experimental setup: A) filter for DNA (0.22 µm membrane Supor), B) filter for nutrient composition GF/F pre-combusted (500 °C, 12 h) filters, C) filters for microscopy (0.22 µm black polycarbonate, Whatman, GE Healthcare, Little Chalfont). D) Layer of nutrient added agar coating all filters. E) Schematic figure showing the colonization tank 120 L, with hanging agar covered filters and pump for water circulation (yellow).

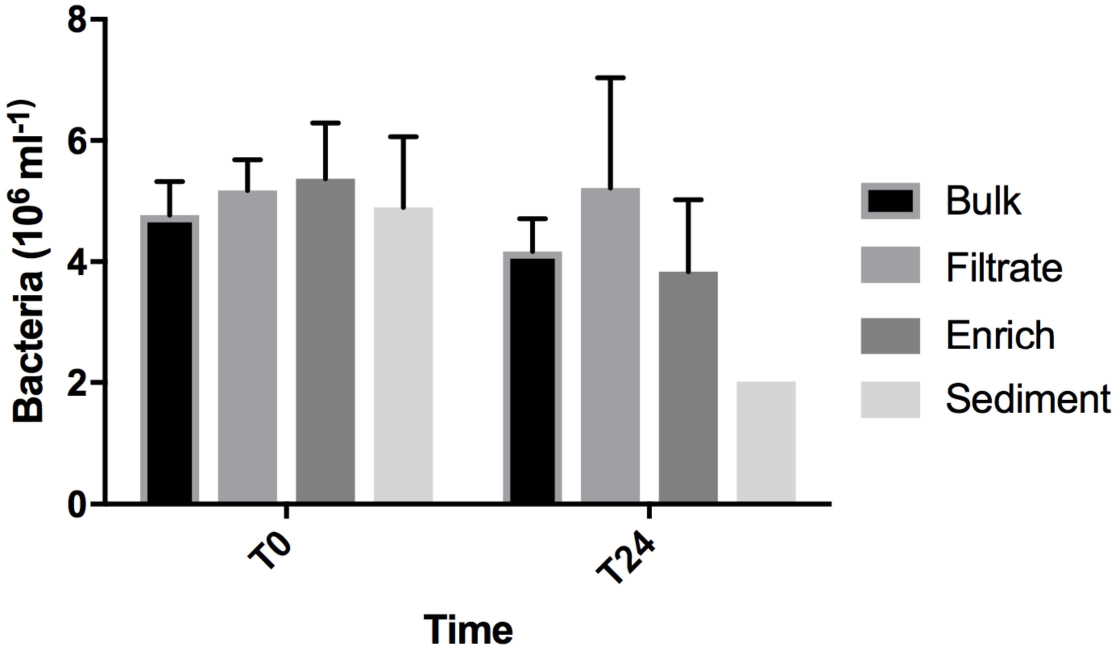

Fig. S2: Bacterial abundance over time in the particle enrichment experiment. n = 3 and n = 4 for T0 and T24, respectively, except for the sediment sample (T24) where only one replicate was available.

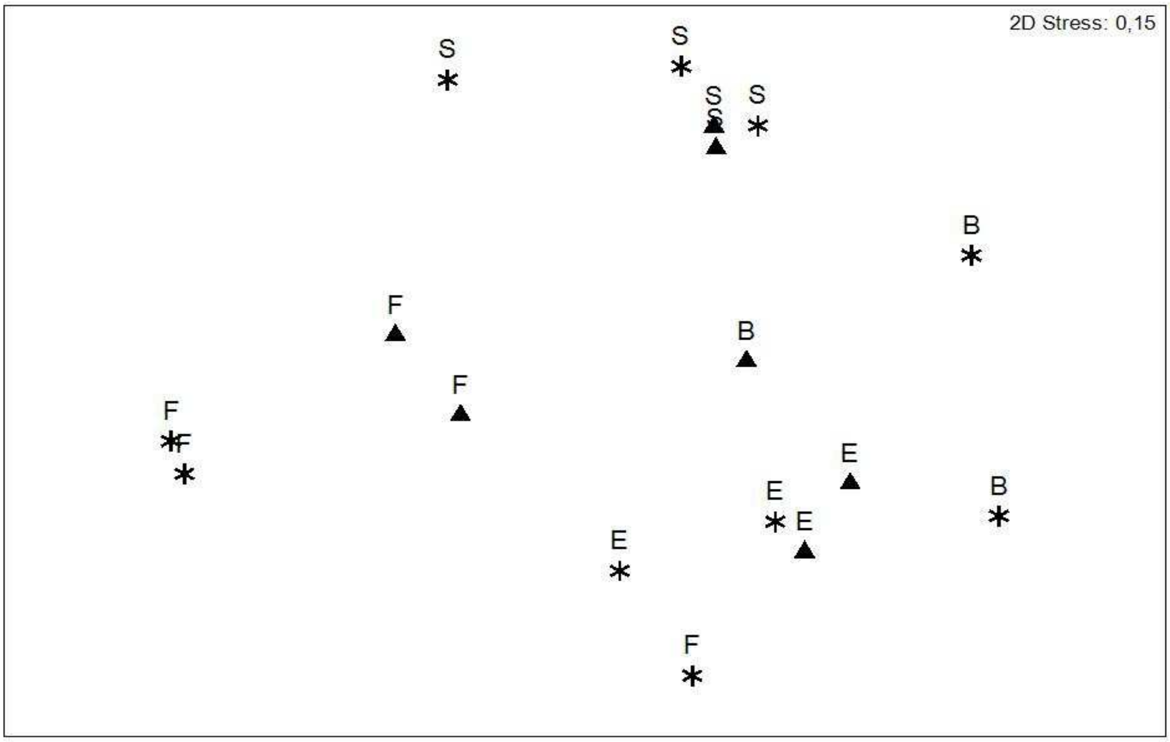

Fig. S3: Non-metric multidimensional scaling plot of Bray Curtis similarity between treatments based on rarefied (n = 5,125) *nifH* gene composition in the particle enrichment experiment. Triangles and stars indicate T0 and T24, respectively, and letters indicate treatment (B = bulk, F = filtrate, E = enriched, S = sediment). Stress 0.15. In some cases, less than the maximum number of replicates per sample is shown since amplification was unsuccessful for single replicates.

Supplementary Tables

Table S1: Nutrient concentrations in the agar used to cover filters in the colonization experiment. Carbon, nitrogen, and phosphorus were added approximately in Redfield ratio (106:16:1, Redfield, 1934). The carbon concentration in the agar is ca. 10 fold higher than average Roskilde Fjord water at the time of sampling (unpublished data, Institute for Bioscience, Aarhus University). The different nutrients were combined so that equimolar concentrations of C, N, and P were added with each source molecule.

Nutrient (Redfield)	Nutrient source	Formula	µM in Agar
Carbon<br>2.5 mM	Glucose	$C_6H_{12}O_6$	83.3
	Galactose	$C_6H_{12}O_6$	83.3
	Mannitol	$C_6H_{14}O_6$	83.3
	Sodium acetate	CH_3COONa	250.0
	Sodium pyruvate	$C_3H_3NaO_3$	166.7
Nitrogen<br>0.37 mM	Potassium nitrate	KNO_3	188.7
	Ammonium chloride	NH_4Cl	188.7
Phosphorus<br>0.02 mM	Sodium phosphate monobasic	NaH_2PO_4	23.6

Table S2: *nifH* (top) and 16S rRNA (bottom) gene primers and sample-specific indexes used for Illumina sequencing in this study.

Name	Gene	Sequence (5' to 3')	Index	Index names	Reference
nifH1-1	nifH	NNNNTAGATCGCTGYGAYCCNAARGCNGA	TAGATCGC	S501	Zehr and McReynolds 1989
nifH1-2	nifH	NNNNTATCCTCTTGYGAYCCNAARGCNGA	TATCCTCT	S503	Zehr and McReynolds 1989
nifH1-3	nifH	NNNNAGAGTAGATGYGAYCCNAARGCNGA	AGAGTAGA	S504	Zehr and McReynolds 1989
nifH1-4	nifH	NNNNGTAAGGAGTGYGAYCCNAARGCNGA	GTAAGGAG	S505	Zehr and McReynolds 1989
nifH1-5	nifH	NNNNACTGCATATGYGAYCCNAARGCNGA	ACTGCATA	S506	Zehr and McReynolds 1989
nifH1-6	nifH	NNNNAAGGAGTATGYGAYCCNAARGCNGA	AAGGAGTA	S507	Zehr and McReynolds 1989
nifH1-7	nifH	NNNNCTAAGCCTTGYGAYCCNAARGCNGA	CTAAGCCT	S508	Zehr and McReynolds 1989
nifH1-8	nifH	NNNNTATAGCCTTGYGAYCCNAARGCNGA	TATAGCCT	S510	Zehr and McReynolds 1989
nifH1-9	nifH	NNNNCCTATCCTTGYGAYCCNAARGCNGA	CCTATCCT	S513	Zehr and McReynolds 1989
nifH1-10	nifH	NNNNGGCTCTGATGYGAYCCNAARGCNGA	GGCTCTGA	S514	Zehr and McReynolds 1989
nifH1-11	nifH	NNNNAGGCGAAGTGYGAYCCNAARGCNGA	AGGCGAAG	S515	Zehr and McReynolds 1989
nifH1-12	nifH	NNNNTAATCTTATGYGAYCCNAARGCNGA	TAATCTTA	S516	Zehr and McReynolds 1989
nifH2-1	nifH	NNNNTCGCCTTAADNGCCATCATYTCNCC	TAAGGCGA	N701	Zehr and McReynolds 1989
nifH2-2	nifH	NNNNCTAGTACGADNGCCATCATYTCNCC	CGTACTAG	N702	Zehr and McReynolds 1989
nifH2-3	nifH	NNNNTTCTGCCTADNGCCATCATYTCNCC	AGGCAGAA	N703	Zehr and McReynolds 1989
nifH2-4	nifH	NNNNGCTCAGGAADNGCCATCATYTCNCC	TCCTGAGC	N704	Zehr and McReynolds 1989
nifH2-5	nifH	NNNNAGGAGTCCADNGCCATCATYTCNCC	GGACTCCT	N705	Zehr and McReynolds 1989
nifH2-6	nifH	NNNNCATGCCTAADNGCCATCATYTCNCC	TAGGCATG	N706	Zehr and McReynolds 1989
nifH2-7	nifH	NNNNCCTCTCTGADNGCCATCATYTCNCC	CAGAGAGG	N708	Zehr and McReynolds 1989
nifH2-8	nifH	NNNNAGCGTAGCADNGCCATCATYTCNCC	GCTACGCT	N709	Zehr and McReynolds 1990
nifH2-9	nifH	NNNNCAGCCTCGADNGCCATCATYTCNCC	CGAGGCTG	N710	Zehr and McReynolds 1991
nifH2-10	nifH	NNNNCGAGTAATADNGCCATCATYTCNCC	ATTACTCG	N721	Zehr and McReynolds 1989
nifH2-11	nifH	NNNNTCTCCGGAADNGCCATCATYTCNCC	TCCGGAGA	N722	Zehr and McReynolds 1989
nifH2-12	nifH	NNNNAATGAGCGADNGCCATCATYTCNCC	CGCTCATT	N723	Zehr and McReynolds 1989
nifH3	nifH	ATRTTRTTNGCNGCRTA			Zani et al. 2000
nifH4	nifH	TTYTAYGGNAARGGNGG			Zani et al. 2000

Name	Gene	Sequence (5' to 3')	Index	Index	Reference
515f-1	prokaryotes	NNNNTAGATCGCGTGCCAGCMGCCGCGGTAA	TAGATCGC	S501	Earth Microbiome Project, Bates et al. 2010
515f-2	prokaryotes	NNNNTATCCTCTGTGCCAGCMGCCGCGGTAA	TATCCTCT	S503	Earth Microbiome Project, Bates et al. 2010
515f-3	prokaryotes	NNNNAGAGTAGAGTGCCAGCMGCCGCGGTAA	AGAGTAGA	S504	Earth Microbiome Project, Bates et al. 2010
515f-4	prokaryotes	NNNNGTAAGGAGGTGCCAGCMGCCGCGGTAA	GTAAGGAG	S505	Earth Microbiome Project, Bates et al. 2010
515f-5	prokaryotes	NNNNACTGCATAGTGCCAGCMGCCGCGGTAA	ACTGCATA	S506	Earth Microbiome Project, Bates et al. 2010
515f-6	prokaryotes	NNNNAAGGAGTAGTGCCAGCMGCCGCGGTAA	AAGGAGTA	S507	Earth Microbiome Project, Bates et al. 2010
515f-7	prokaryotes	NNNNCTAAGCCTGTGCCAGCMGCCGCGGTAA	CTAAGCCT	S508	Earth Microbiome Project, Bates et al. 2010
515f-8	prokaryotes	NNNNTATAGCCTGTGCCAGCMGCCGCGGTAA	TATAGCCT	S510	Earth Microbiome Project, Bates et al. 2010
515f-9	prokaryotes	NNNNCCTATCCTGTGCCAGCMGCCGCGGTAA	CCTATCCT	S513	Earth Microbiome Project, Bates et al. 2010
515f-10	prokaryotes	NNNNGGCTCTGAGTGCCAGCMGCCGCGGTAA	GGCTCTGA	S514	Earth Microbiome Project, Bates et al. 2010
515f-11	prokaryotes	NNNNAGGCGAAGGTGCCAGCMGCCGCGGTAA	AGGCGAAG	S515	Earth Microbiome Project, Bates et al. 2010
515f-12	prokaryotes	NNNNTAATCTTAGTGCCAGCMGCCGCGGTAA	TAATCTTA	S516	Earth Microbiome Project, Bates et al. 2010
806r-3	prokaryotes	NNNNTTCTGCCTGGACTACHVGGGTWTCTAAT	AGGCAGAA	N703	Earth Microbiome Project, Bates et al. 2010
806r-4	prokaryotes	NNNNGCTCAGGAGGACTACHVGGGTWTCTAAT	TCCTGAGC	N704	Earth Microbiome Project, Bates et al. 2010
806r-5	prokaryotes	NNNNAGGAGTCCGGACTACHVGGGTWTCTAAT	GGACTCCT	N705	Earth Microbiome Project, Bates et al. 2010

Table S3: Nearest relatives of predominant *nifH* OTUs from particle enrichment experiment (PE) and colonization (CT0) experiment. If OTUs from the different experiments were identical they are placed in the same box. Searches were conducted at the nucleotide level (BLAST) or the protein level (BLASTX; marked by *). *nifH* gene subclusters were assigned according to Frank et al. (2016).

OTU PE/CT0	BLAST	Source	Description	ID	Identity %	*nifH* Cluster	Taxonomy
OTU_1/ CT0_2	BAL376	Baltic Sea, surface_	BAL376 clone 507_3b_B2	KC140352	100	1G	Unassigned
OTU_2 / CT0_5	Alcaligenaceae	Bottled mineral water	*Alcaligenaceae derxia* (França et al. 2016)	KT964778	99.1	1K	Beta-Proteobacteria
OTU_3	uncultured	Cordgrass rhizosphere	Nitrogenase reductase	AF216899	96	3H	Putative anaerobe
OTU_5	uncultured	North Sea sediment		KP959536	100	1A	Unassigned
OTU_6	uncultured	Marine microbial mats	Uncultured bacterium clone 07-II.4 nitrogenase (*nifH*) gene, partial cds	GU193531	99.7	3E	Putative anaerobe
OTU_7	uncultured	Marine microbial mats	Uncultured bacterium clone 08-III.17 nitrogenase (*nifH*) gene, partial cds	GU193899	99	3J	Putative anaerobe
OTU_13	uncultured	Sediment	Uncultured bacterium clone from oil contamination	DQ078033	96	1G	Unassigned
OTU_24	*Desulfosarcina cetonica**	Flooded oil stratum Caspian Sea	*Desulfosarcina cetonica* Strain: JCM 12296	WP_054697558.1	96	3H	Putative anaerobe

OTU 51 /CT0_1	Uncultured*	Seawater of Knorr Expedition	Nitrogen-fixing bacterium clone	HM801708	99	1G	Unassigned
CT0_3	uncultured	Narragansett Bay	Marine sediment mesocosms	JN645307	100	1G	Unassigned
CT0_6	*Amphritea atlantica**	Atlantic Ocean: Logatchev hydrothermal vent	Strain: DSM 18887	SEQ47070.1	100	1G	Unassigned
CT0_7	*Paraburkholderia tuberum**	Symbiont of *Mimosa pudica*	Strain:STM 4252	CCJ09519.1	100	1K	Beta-Proteobacteria
CT0_12	*Desulfopila aestuarii**	estuarine sediment	Strain: DSM 18488	WP_073612834.1	99	3E	Putative anaerobe

Table S4: Bacterial colonization of filter surfaces over time.

Time (h)	Abundance (Cells mm^{-1})	Colonization rate (Cells mm^{-2} h^{-1})
0	52	
15	153	7
40	4,613	12
80	8,038	189
140	41,424	556
210	87,545	659
305	125,257	397
400	136,686	120

Table S5: Morisita-Horn dissimilarity matrix based on 16S rRNA genes obtained from the colonization experiment. Index goes from 0 (identical) to 1 (completely different). Rep. is the difference between replicates (n = 3 for all).

16S rRNA	Rep.	T40F	T40W	T80F	T80W	T140F	T140W	T300F	T300W	T400F
T40F	0.028									
T40W	0.010	0.654								
T80F	0.032	0.113	0.791							
T80W	0.013	0.806	0.546	0.865						
T140F	0.016	0.304	0.755	0.291	0.828					
T140W	0.007	0.855	0.741	0.837	0.731	0.351				
T300F	0.021	0.460	0.806	0.471	0.870	0.096	0.360			
T300W	0.010	0.503	0.740	0.449	0.815	0.433	0.587	0.517		
T400F	0.015	0.483	0.816	0.492	0.886	0.121	0.377	0.020	0.511	
T400W	0.074	0.380	0.715	0.380	0.828	0.320	0.704	0.343	0.205	0.324

Table S6: Nearest relatives of the operational taxonomic units (OTUs), obtained from the colonization experiment, based on 16S rRNA gene sequencing. OTU`s shown here accounted for >80% of all reads in each sample. On the left side is the taxonomy assigned according to the SILVA database and on the right side is environment of origin (source), GenBank accession number (ID), and percent similarity to closest marine hit (Sim.). The far right column indicates whether the nearest relative was obtained from water (F = free-living) or from a surface (S).

Assigned by SILVA database				BLAST Hit			Assigned
OTU	Family	Genus	Species	Source	ID	Sim. (%)	Free-liv./Surface
OTU_4	Rhodobacterales	Rhodobacteraceae	Loktanella	Seawater	KF911340	100	F
OTU_9	Micrococcales	Microbacteriaceae	Candidatus Aquiluna	Seawater, 2 m depth	FR648224	100	F
OTU_14	Burkholderiales	Alcaligenaceae	Achromobacter	Chesapeake Bay	EF471609	99	F
OTU_8	Alteromonadales	Alteromonadaceae	Glaciecola	Seawater	KR077364	100	F
OTU_22	Oceanospirillales	Oceanospirillaceae	Pseudospirillum	Baltic Seawater	KC899249	100	F
OTU_20	Flavobacteriales	Flavobacteriaceae	NS3a marine group	Surface seawaters	KU173779	100	F
OTU_19	SAR11 clade	Pelagibacteriales	Uncultured marine	Surface mucus	KU243169	100	S

			bacterium				
OTU_23	SAR11 clade	Pelagibacteriales	Uncultured marine bacterium	Chesapeake-Delaware Bay water samples	JX529400	100	F
OTU_34	Micrococcales	Microbacteriaceae	Candidatus Limnoluna	Seawater, 2 m depth	FR648015	100	F
OTU_33	Oceanospirillales	Oceanospirillaceae	Uncultured	Artificial surfaces submerged in marine water	EF215802	100	S
OTU_15	Oceanospirillales	Oceanospirillaceae	Oleispira	Seawater	KM277246	100	F
OTU_695	Unknown			No close hit			
OTU_12	Flavobacteriales	Flavobacteriaceae	Uncultured	Seawater;	JQ198834	98	F
OTU_6	Oceanospirillales	Oceanospirillaceae	Marinomonas	Ciliate cell	KU524802	100	S
OTU_3	Rhodobacterales	Rhodobacteraceae		Intertidal beach	GU061240	100	S
OTU_10	Alteromonadales	Pseudoalteromonadaceae	Pseudoalteromonas	OMZ water	KY604938	100	F

				column			
OTU_18	Sphingobacteriales	Saprospiraceae	Aureispira	Shell surface	JN987168	100	S
OTU_264	Rhodobacterales	Rhodobacteraceae	uncultured	Macroalgal surface	GU451450	100	S
OTU_11	Oceanospirillales	Oceanospirillaceae	Reinekea	Oil-contaminated seawater,	KJ139653	100	F
OTU_27	Oceanospirillales	Oceanospirillaceae	Oceanobacter	Sediment	KC476293	100	S
OTU_13	Alteromonadales	Colwelliaceae	Colwellia	Deep sediment	LC138576	100	S
OTU_17	Alteromonadales	Alteromonadaceae	Glaciecola	*Ascophyllum nodosum*	LN881333	100	S
OTU_144	Rhodobacterales	Rhodobacteraceae	Pacificibacter	Biofilm formation	GQ274155	99	S
OTU_7	Oceanospirillales	Oceanospirillaceae	Neptunomonas	Seawater_Ireland	JQ436093	100	F
OTU_5	Alteromonadales	Colwelliaceae	Colwellia	Seawater_Ireland	JQ436093	100	F
OTU_16	Flavobacteriales	Flavobacteriaceae	Maribacter	Shallow-sea	KX022625	100	S

				hydrothermal			
OTU_712	Rhodobacterales	Rhodobacteraceae	Uncultured	Tidal flat	KC195795	98	S
OTU_2	Rhodobacterales	Rhodobacteraceae		Corrosion biofilm	LT549343	100	S
OTU_1	Flavobacteriales	Flavobacteriaceae	Uncultured	Surface of algae	HM437512	99	S

Table S7: Morisita-Horn dissimilarity matrix based on *nifH* genes from samples obtained over time in the colonization experiment; 0 – 400 h. The index goes from 0 (identical) to 1 (completely different). Rep. is the difference between replicates. F and W indicate filter and water, respectively. Numbers of replicates are given in parentheses.

NifH	Rep.	T0W (1)	T80F	T140W	T300F	T300W	T400F
T80F (1)		1.000					
T140W (2)	0.676	0.990	0.962				
T300F (2)	0.027	0.983	0.927	0.788			
T300W (3)	0.002	0.992	0.979	0.803	0.016		
T400F (3)	0.000	0.993	0.978	0.813	0.021	0.002	
T400W (3)	0.000	0.993	0.979	0.816	0.023	0.003	0.001

Reference List

Bates S. T., Berg-Lyons D., Caporaso J. G., Walters W. A., Knight R., Fierer N. (2010) Examining the global distribution of dominant archaeal populations in soil. ISME J 5:908-917

Frank I. E., Turk-Kubo K. A., Zehr J. P. (2016) Rapid annotation of nifH gene sequences using classification and regression trees facilitates environmental functional gene analysis. Environ. Microbiol. Rep. 8:905-916

Redfield, A. C. (1934) *On the proportions of organic derivatives in sea water and their relation to the composition of plankton*. University press of Liverpool.

Zani, S., Mellon, M. T., Collier, J. L., and Zehr, J. P. (2000). Expression of nifH genes in natural microbial assemblages in Lake George, New York, detected by reverse transcriptase PCR. Appl. Environ. Microbiol. 66:3119-3124.

Zehr JP, McReynolds LA (1989) Use of degenerate oligonucleotides for amplification of the *nifH* gene from the marine cyanobacterium *Trichodesmium thiebautii*. Appl.Environ.Microbiol. 55:2522-2526
